# The Migration and Invasion of Oral Squamous Carcinoma Cells: Matrix, Growth Factor and Signalling Involvement

**DOI:** 10.3390/cancers13112633

**Published:** 2021-05-27

**Authors:** Ian R. Ellis

**Affiliations:** Department of Cell and Molecular Biology, School of Dentistry, University of Dundee, Dundee DD1 4HR, UK; i.r.ellis@dundee.ac.uk

The link between the migration of cancer cells and the spread of cancers has been established for many years. Tumour invasion and metastasis was labelled as one of the original Hallmarks of Cancer in the seminal paper written by Hanahan and Weinberg at the turn of the century [1]. The definition was further updated in 2011 to be described as the activation of invasion and metastasis [2]. This update re-introduced the fact that the tumour microenvironment was again mainstream thinking due to the re-evaluation of the Paget’s “seed and soil” hypothesis which dated back to 1889 [3]. Therefore, it is not just the cancer cells themselves that are important, but it is the interactions of the tumour and the stroma that are vital. Studies into these interactions of tumour and “host” have been integral in investigating the role of growth factors (and their receptors), extracellular matrix molecules (and their receptors) and cell signalling pathways and the crosstalk between all these factors. This has led to many initially interesting findings in the laboratory not living up to expectation when arriving in the clinic. With the advent of new techniques and in combination with tried and tested methods new insight will hopefully lead to the introduction of therapies from which patients will see a benefit.

The tumour microenvironment (TME) is made up of complex mixture of tumour cells and stromal derived cells but also containing a modified extracellular matrix (ECM). The ECM is the major structural component of the body comprised of proteins, glycoproteins, glycosaminoglycans and other polysaccharide in a network which would be able to host tumour cells (seed and soil theory) and control whether the tumour will be able to grow and metastases or not be able to move freely through a tissue [3,4]. Modulation of this ECM is essential for the migration of tumour cells [5].

Modulation of the ECM can also initiate the release of cytokines and growth factors that can stimulate pathways that allow for cells to migrate whether in single cells or as sheets [6]. Growth factors have long been thought to have both a positive and a negative effect on cancer cells. Transforming growth factor beta 1 (TGFβ-1) has both tumour suppressor and promoter characteristics: TGFβ-1 initially inhibits the progression of cells into a cancerous state but once these cells are activated TGFβ-1 becomes a cancer promoter [7]. This was further supported by work by the Schor group (2012) where they reported that the ECM cells on which were plated was important. Cells plated on a broken-down matrix (gelatine) responded to TGFβ-1 by switching on migration stimulating factor (MSF) expression. This only being able to be down-regulated when the activated cells were plated onto 3D collagen matrix and re-exposed to TGFβ-1 [8].

Growth factors and ECM can both modulate cell signalling pathways. Analysis of genomes from Head and Neck Cancer patients (*n* = 279 where 172 have a tumour of the oral cavity and most likely to be oral squamous carcinoma) indicates several signalling pathways such as Akt/PI3K that are up-regulated in these patients [9,10]. Head and Neck cancer squamous carcinoma cell invasion was reviewed comprehensively by Kramer et. al., they reported that all the components of the tumour microenvironment are involved in enabling tumour cell motility [11]. This illustrates that oral squamous carcinoma cells follow the same patterns as other tumours in implicating, the interaction of matrix, growth factors and signalling pathways. More recent data has followed specific lines of enquiry into TGFβ-1, EGF, EGFR and Akt [12], as well as NGF and Akt [13].

Therefore, we welcome manuscripts describing cell biological, immuno-histochemical and biochemical studies of factors affecting cell migration of Oral Squamous Cell Carcinoma (OSCC) and how this translates into the clinical environment. We are also interested in research involving the studies of novel therapies or novel therapeutic approaches which may block cell migration and leading to possible new treatments.
